# Novel Method of Monitoring Trace Cytokines and Activated STAT Molecules in the Paws of Arthritic Mice using Multiplex Bead Technology

**DOI:** 10.1186/1471-2172-11-55

**Published:** 2010-11-12

**Authors:** Lily D Lu, Kristine L Stump, Matthew M Seavey

**Affiliations:** 1 Worldwide Discovery Research, Cephalon, Inc., West Chester, Pennsylvania 19380, USA

## Abstract

**Background:**

The use of mouse models to study human disease provides useful data that can provide support for research projects or an existing drug discovery program. How well a model recapitulates the human condition and the ease and reproducibility of data collected will determine how much confidence a scientist can place on results obtained. Designing new treatments for rheumatic diseases, such as rheumatoid arthritis (RA), requires complex immunocompetent models that depend on intricate cytokine networks. Using local cytokines, signal transduction and transcription factor molecules as potential biomarkers to monitor disease and treatment efficacy is the best method to follow the progression of tissue damage and repair when testing an unknown compound or biologic. Described here in this report, a novel method for the non-enzymatic extraction and measurement of cytokines and signal transducers and activators of transcription (STAT) molecules using Luminex^® ^bead array technology in two different mouse models for human RA - collagen antibody-dependent arthritis (CAIA) and collagen-induced arthritis (CIA).

**Results:**

Dynamic expression of several pro-inflammatory cytokines responsible for promoting disease augmentation overtime were monitored, such as IL-1β, TNFα, IL-6 and IL-12, locally in the paws of affected animals directly *ex vivo*. Local cytokine responses could be matched with serum cytokine levels and joint pathology results. In addition, STAT1, 3, and 5a/b activation status could be monitored with confidence using specifically formulated extraction buffer that protected the phosphorylation site. STAT3 activation followed paw swelling and cytokine levels in both models and correlates of disease could be ablated upon treatment with dexamethasone. Here reported a novel method of extracting joint fluid from the paws of inflamed mice coupled with powerful multiplex bead technology allowing us to measure cytokine responses, pharmacodynamic markers such as STATs and pharmacokinetic analysis of dosed agent all from the same sample directly *ex vivo*.

**Conclusions:**

This method is powerful in that it is applicable to multiple autoimmunity model types, streamlines *ex vivo *readouts in a high-throughput manner, and allows multiplexing providing the investigator with an array of options and possible analytes when developing preclinical animal models to support drug discovery efforts in the search for new treatments for rheumatic diseases.

## Background

Rheumatoid arthritis (RA) is a debilitating disease that results from chronic inflammation within the synovial lining and lumen of multiple joints. Overtime this can result in bone resorption and shortened life span among those effected [1,2]. Using animal models to recapitulate human disease is commonly employed to both study disease mechanisms and for the discovery of new treatments. One of the two most commonly used mouse models for human RA is the acute antibody driven collagen type II (CII) dependent inflammatory response produced upon the intravenous (i.v.) transfer of anti-CII antibodies, (i.e., collagen-antibody-induced-arthritis or CAIA). The second and more physiological counter-part model involves overcoming endogenous tolerance to CII. Administration of self-protein, CII, along with an adjuvant, heat killed Mycobacterium suspended in mineral oil, or complete Freud's adjuvant (CFA) provides a powerful pro-inflammatory response driving potent anti-CII CD4^+ ^T-cell helper-1 (Th1) and T-helper-IL-17 (Th17) cellular and anti-CII antibody responses. This model referred to as collagen-induced arthritis (CIA) recapitulates several of the more common phenotypes observed in human disease such as flares, chronic inflammation and bone resorption often leading to rheumatoid arthritis [3].

Monitoring peripheral blood cytokine levels in these models can be performed using various assays from ELISAs to multiplex bead analysis. However, several studies have clearly demonstrated the importance of studying the local immune responses at the site of disease or insult as being the most reflective of disease progression and/or appropriate response for treatment [4-6]. The fundamental cornerstone of drug discovery is the ability to select a viable target and being able to intercept that target as to modulate disease activity in a specific fashion. Monitoring local cellular and biochemical responses at the tissue site of disease is crucial for predicting future disease outcomes and thus drug treatment efficacy.

For CIA and CAIA models, local pro-inflammatory cytokines and related signaling molecules and/or transcription factors such as Signal Transducers and Activators of Transcription (STATs) parallel disease progression [4,6]. Increases or decreases in such molecules may predict treatment efficacy and rate of response [5,7,8]. From this logic a novel methodology was designed to sample external cellular milieu directly from cryopreserved paws and limbs of arthritic mice and quantitate trace cytokines and STAT activation (i.e., phosphorylation) directly *ex vivo*. Both molecular readouts followed the disease model and were predictive of local responses. This method will provide the investigator with a way to follow cytokine responses with signal transduction cascades and compound levels in a single tissue from a single processed sample in a high-throughput manner.

## Methods

### Animals

Mice used were Harlan DBA/1 mice (strain: *DBA/1OlaHsd*). Mice were all aged matched, six to eight weeks from start of experiment. All animals were maintained on a 24 hour light/dark cycle (on at 7 AM/off at 7 PM), with food and water available *ad libitum*. All experimental animal procedures were approved by and in accordance to the regulations of the Institutional Animal Care and Use Committee (IACUC) of Cephalon, Inc.

### Collagen-Antibody Induced Arthritis (CAIA) Model

Acclimated mice were randomized, pre-bled and measured for baseline inflammation before any studies were initiated. On day 0, DBA/1 female mice were injected i.v. with 100 μl of 1.5 mg of a 10 mg/ml cocktail arthritogeneic monoclonal antibodies directed against different epitopes of collagen type II (Chondrex, Redmond, WA) suspended in saline. On day 3, mice were injected with 100 μl of 50 μg of *E. Coli *0111:B4 (0.5 mg/ml stock) LPS (Chondrex, Redmond, WA) i.p. and provided with gel food during recovery. Two days post-LPS treatment monitoring of arthritic paws began, peak arthritis usually occurred 2-3 days following LPS treatment. Before starting treatment mice were randomized, grouped, scored, ear tagged and pre-bled to determine baseline disease. Mice that exhibited a clinical score greater than "1" in each limb were considered arthritic and entered the study. Arthritis clinical score tables can be found described elsewhere [3], briefly, score '0' = no evidence of erythema and swelling; score '1' = erythema and mild swelling confined to the tarsus; score '2' erythema and mild swelling extending from the ankle to mid-foot; score '3' erythema and moderate swelling extending from the ankle to the metatarsal joints; score '4' erythema and severe swelling encompass the ankle, foot and digits. Treatment with Dexamethasone (Hanna's Pharmaceutical Supply, Wilmington, DE) at 1.5 mg/kg was administered i.p. three times a week in saline, vehicle consisted of saline alone. Dexamethasone treatment continued from the peak of inflammation until the end of the experiment. Mice were cheek bled for serum sample collection for cytokines and antibodies throughout the experiment. All samples were kept at -80°C until ready to assay. At about 4 weeks mice were harvested for spleen, plasma and arthritic paws for STAT and cytokine quantitation and histological analysis. For earlier validation studies we ended studies around 3-4 weeks post induction and removed paws at various different time points to measure cytokine levels overtime.

### Collagen-Induced Arthritis (CIA) Model

Acclimated mice were randomized and pre-bleed and measured for baseline inflammation before any studies were initiated. On day 0, DBA/1 female mice were given a primary immunization with equal volumes of bovine collagen type II (CII), 2 mg/ml stock in 0.05 M acetic acid, (Chrondrex, Redmond, WA) suspended in a CFA, 5 mg/ml (Chondrex, Redmond, WA) emulsion yielding an injection of 100 μg of CII at 100 μl into the base of the tail intradermally (i.d.). At day 21 post primary immunization, the mice were rechallenged with CII emulsified in IFA (Thermo Scientific, Rockford, IL) s.c. on the flank of the mouse. On day 28 each mouse received an i.p. injection of *E. coli *0111:B4 (0.5 mg/ml stock) LPS (Chondrex, Redmond, WA) at 10 μg in 100 μl of saline (25-50 μg of LPS was reduced to 10 μg per mouse to reduce mortality associated with endotoxic shock). Around day 35 the mice begin to show signs of paw inflammation and disease. Before starting treatment mice were randomized, grouped, scored, ear tagged and pre-bled to determine baseline disease. Mice that exhibited a clinical score greater than "1" in each limb was considered arthritic and entered the study. Arthritis clinical score was the same as that used to score CAIA model. Treatment with Dexamethasone (Hanna's Pharmaceutical Supply, Wilmington, DE) at 1.5 mg/kg was administered i.p. three times a week in saline, vehicle consisted of saline alone. Dexamethasone treatment continued from the peak of inflammation until the end of the experiment. Mice were cheek bled for serum sample collection for cytokines and antibodies throughout the experiment. All samples were kept at -80°C until ready to assay. At about day 60 mice were harvested for spleen, plasma and arthritic paws for STAT and cytokine quantitation and histological analysis.

### Histology

Front and hind paws (including carpus and tarsus) were removed from the body of the animal. Skin from the ends of the digits removed and the metatarsal region skin perforated using surgical scissors to allow full decalcification. Samples followed a decalcification procedure using formic acid as described elsewhere [9,10]. After 7-10 days of decalcification samples were washed for 2 hours in distilled water and stored in 70% ethanol at 4°C until ready to be processed. Samples were paraffin embedded, sectioned and stained with Haemalytoxin and Eosin or Safranin-O stain. Safranin-O stain was performed via manufacteurer instruction (Poly Scientific, R+D). All histology work was performed at the Wistar Institute (Philadelphia, PA). Images were collected using an Olympus BX50 scope with an Olympus DP70 camera and Olympus LabSens software (2009). A total of five mice from each group were analyzed via histopathology, representative images are shown for each group tested.

### Western Blot Analysis

Samples were heated at 70°C for 10 minutes and loaded onto a NuPAGE 10% Bis-Tris Gel, 1.5 mm, 15 well (Invitrogen, no. NP0316BOX). The molecular weight marker used was a BioRad Kaleidoscope prestained standard (BioRad, no. 161-0324). Run conditions were 150 volts for 1 hour at RT. Using a semi-dry transfer apparatus (BioRad, no. 170-3940) proteins were transferred into a nitrocellulose membrane. Primary antibody diluted at 1:1000 in 5% milk for phosphorylated STAT3 pY705 (Cell Signaling, no. 9131) and total STAT3 (Cell Signaling, no. 9132) diluted to 1:1000 in 5% BSA in TBS were incubated overnight at 4°C with 5% milk in TBS plus 0.05% Tween-20. Band sizes for STAT3α and STAT3β are 79 kDa and 86 kDa respectively. Anti-rabbit IgG, HRP-linked secondary antibody was used at 1:2000 (Cell Signaling, no. 7047). Detection was performed using SuperSignal West Pico Chemiluminescent Substrate (Pierce, no. 34077). Densitometry was performed using GelPro analyzer 3.1 software.

### Measurement of Serum Anti-Collagen type I and type II autoantibodies

Serum was collected and stored at -80°C until use. Thawed samples were analyzed by an in-house generated collagen type II and type I ELISA method. This procedure can be found elsewhere [11], briefly each well of a 96-well plate is coated with 50 μl of 5 μg/ml of collagen type I (Chondrex, no. 1006) or collagen type II (Chondrex, no. 2016) in BBS (0.025 M Na_2_B_4_O_7_-10H_2_O, 0.01 M H_3_BO_3_, 0.075 M NaCl, pH 8.4) buffer overnight at 4°C. Plates were washed with BBS plus 0.1% Tween-20 three times before proceeding. Standard curves were generated using purified mouse anti-collagen type I or type II antibody, serum was added at 50 μl per well, incubated at RT for 1 hour then washed with BBS plus Tween-20. After washing, 50 μl of 1 μg/ml of a rabbit anti-mouse HRP-Fab fragment is added as a detection antibody (Rockland, no. 811-1302) and incubated at RT for 45 minutes. Plates were washed four times with BBS plus Tween-20, then 100 μl of TMB substrate is added and the reaction is stopped using 100 μl of 1 M H_2_SO_4_. The plates were read at 450 nM with a reference length of 570 nM.

### Measurement of Serum Cytokines via Multiplex Luminex^® ^Beads Assays

Frozen plasma at -80°C was thawed on ice, vortexed then centrifuged for 10 minutes to remove debris and aggregates. A total of 25-50 μl of serum was used for Luminex^® ^assays following the manufacture's instruction. Ten different mouse cytokines were measured using the mouse cytokine 10-plex bead kit (Invitrogen, no. LMC0001). Briefly, filter plates (Millipore, no. MAIPSWU10), were pre-wet with 200 μl of wash solution (kit component) and 25 μl of beads were added per well. Serum samples were diluted and a total volume of 50 μl was added per well (i.e., 25 μl of sample serum plus 25 μl of assay diluent). Plates with beads were incubated for 2 hours at RT on an orbital shaker in the dark. At the end of the incubation the plate(s) were washed twice in buffer, secondary biotinylated antibody was added at a 1:10 dilution, 100 μl, in biotin diluent provided with the kit. Plates were incubated at RT for 1 hour in the dark then washed twice in buffer. Streptavindin in assay diluent was added at 100 μl per well, then incubated at 30 minutes at RT in the dark. Plate was washed three times then we added 100 μl of wash solution and agitated the plate for 2-3 minutes at RT in the dark. Plates were ran immediately on a Luminex xMAP 200 unit with data acquisition and analysis software (Invitrogen, no. MAP0200). All bead washing was performed using a vacuum manifold unit (Pall, no. 5017).

### Measurement of Paw Cytokines and Phospho-STATs via Multiplex Luminex^® ^Bead Assays

The front (including carpus) and back (including tarsus) right paws were collected and frozen in dry-ice cooled isopentane and stored at -80°C until use. The paws were cut to 2 mm × 2 mm segments and kept on dry-ice in round bottom polypropylene tubes (BD Falcon, no. 352059). Seven hundred microliters tissue extraction buffer containing protease inhibitor cocktail (Calbiochem, no. 539136) and Halt phosphatase inhibitor cocktail (Thermo scientific, no. 78420) or Roche phosphatase inhibitor cocktail (Roche, no. 04906837001) in tissue extraction reagent I (Invitrogen, no. FNN0071) were added to each sample. Samples were homogenized frozen using a PT 10-35 Polytron homogenizer (VWR, no. 97036-082). After homogenization the sample was centrifuged at 4°C for 2000 × g for 10 minutes, supernatant was re-centrifuged at 4°C at maximum speed, 14,000 × g, for 15 minutes. Supernatants were carefully removed to avoid picking up the top layer of lipids/adipose debris. Protein concentration was adjusted to 3 mg/ml using a BCA protein assay (Pierce, no. 83228). A minimum of 25 μl of this extraction is used in the above described protocol using the mouse cytokine 10-plex bead kit (Invitrogen, no. LMC0001) or phophorylated STAT1, 3, 5a/b, 3-plex kit (Invitrogen, LHO0005).

### Compound quantification (PK, Pharmacokinetics)

Plasma and protein extraction from paw and spleen samples were submitted for quantitative analysis to determine the respective compound concentrations. Blood samples were collected into heparinized tubes and placed on wet ice until centrifuged (16,000 × g, 5 minutes) to separate the plasma. Supernatant was collected and stored at -20°C pending analysis. At the time of analysis, two volumes of cold acetonitrile containing an internal standard (alprenolol) was added to each sample which was then vortexed and centrifuged. The supernatant was removed, placed into an auto-sampler vial and the amount of compound present in the samples was analyzed by liquid chromatograph/mass spectrometry (LC-MS-MS). The concentration of compound in the samples was quantified against a mouse plasma standard curve made via serial dilution in a concentration range from 5 to 20,000 ng/mL. Samples containing concentrations greater than 10% above the top of the standard curve were diluted 1:10 with acetonitrile. The limit of detection for plasma, paw and spleen were < 10 ng/ml.

### Statistical Analysis

All ELISA or Luminex^® ^assays were analyzed using linear regression curves to determine concentration of analyte following data acquisition. Mann-Whitney non-parametric, 2-way ANOVA or the Student's t-test were used for data analysis as noted in figure legends depending on the experiment and tested hypothesis. A p-value less than 0.05 was considered significant. Statistical software used was Graph Pad Prism (vs. 5.01, 2007), calculations were performed using Microsoft Office Excel (Professional, 2003).

## Results

### Detection of Trace Cytokines in Two Models of Rheumatoid Arthritis

Reproducing human disease in mouse models provides the investigator with a reproducible and controlled system whereby experiments can be performed rapidly to test various reagents, therapeutics and methods. The two most commonly used RA models for drug discovery include the collagen type II antibody-induced arthritis model (CAIA) and the more conventional collagen type II-induced arthritis model (CIA). The CAIA model is a great system to study human RA in a drug discovery setting as it requires little set-up time and a meaningful result can be obtained in only a few weeks. However, one of the major disadvantages of using this model is that since there is not a sufficient cellular anti-self immune response generated, many of the classical RA promoting cytokines are undetectable in the serum of diseased mice [12,13]. Much of the immune response is driven by antibody-dependent-cellular-cytotoxicity (ADCC) at the site of disease, the synovial joint. Transient but significant cytokine accumulation would be expected in the local tissue and possibly the draining lymph node(s). For drug discovery efforts it is best to chose a model that both recapitulates the human condition but also provides sufficient readouts in a relatively short time frame. The modulation of pro-inflammatory, disease-promoting cytokines, such as TNFα, IL-1β and IL-12, using either biologics or small molecule inhibitors is crucial to controlling RA, and many trials are still in progress demonstrating this concept [14-17].

**Figure 1:**
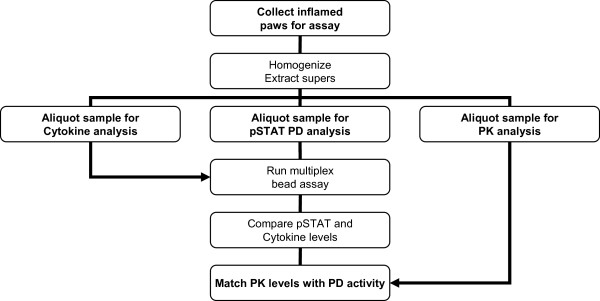
**Paw Cytokine Extraction Method Flow Sheet**. As the illustration demonstrates, paws are selected for processing, snap frozen and stored at -80°C until ready to be processed. When ready, samples are placed on dry ice and homogenized frozen using prepared tissue extraction buffer. An aliquot is used for Luminex^® ^cytokine analysis, phosphorylated STAT (pSTAT) pharmacodynamic (PD) Luminex^® ^analysis and another aliquot used for pharmacokinetic (PK) analysis. After all three analyses are finished; the PD data is compared to the functional cytokine readouts. PK data should always be compared against the functional PD data to see if pharmacological saturation of the target has been achieved. Thus, multiple cytokines and intracellular signaling molecules can be monitored using multiplex bead technology to better predict local disease outcomes and help design more targeted therapies.

**Figure 2:**
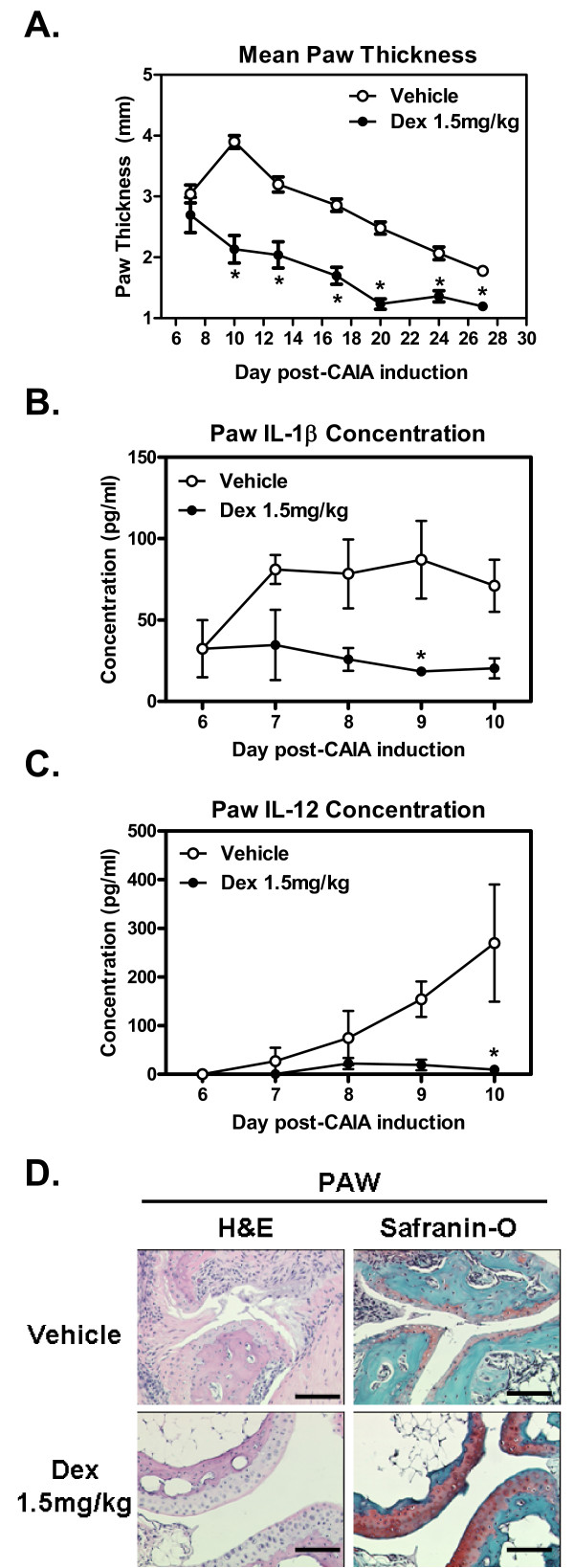
**Paw Cytokines Follow Disease Progression in CAIA Mouse Model**. Female DBA/1 mice were injected i.v. with anti-collagen type II antibodies and given a LPS booster injection three days after the antibody transfer. This combination of antibodies and adjuvant provides a potent and rapid, but self-limiting, antibody dependent arthritis. **(A) **Disease is established and mice enter the study when specific criteria are met (see materials and methods). Dexamethasone significantly reduced mean paw thickness (averaged from individually measured paws - front, back, right and left), *p < 0.01 versus vehicle control group, N = 10 per group. **(B) **Paw IL-1β concentration sampled in 3 mg of total protein prepared as described in the materials and methods. As expected from the paw swelling, dexamethasone reduces cytokine levels compared to the vehicle control group, *p < 0.05. **(C) **Paw IL-12 slowly increases overtime as disease progresses, dexamethasone treatment significantly reduces paw cytokine levels, *p < 0.05, N = 5 per group. **(D) **Histological analysis of paw swelling using H&E and Safranin-O stains (carpus shown). Vehicle shows leukocytic infiltrates, matrix degradation and osteolysis, dexamethasone treated mice compared to non-diseased animals (data not shown), N = 5 mice per group, representative images shown, 4× magnification. All graphs show Mean ± SEM.

Since serum cytokines are difficult to measure using this model [12,13] and since the most reflective disease activity can be found locally in the paw [18] building an extraction assay whereby cytokines are collected from inflamed paws and read on a multiplex bead assay using the powerful Luminex^® ^technology would be greatly beneficial. As illustrated in the method schematic flow chart (Figure 1), front and back paws are collected and cut into small pieces (2 mm × 2 mm) then snap frozen and stored at -80°C until ready for processing. Keeping everything on dry-ice, samples were homogenized and cellular/joint extracts were analyzed for either cytokine or phosphorylated STAT activation. Using Dexamethasone at 1.5 mg/kg body weight we were able to validate our method modulating the local cytokine milieu using a standard-of-care option, (i.e., glucocorticoid treatment). Established arthritis was generated and glucocorticoid, dexamethasone, was given three times a week, i.p., and reduced paw swelling measured via a digital caliper (Figure 2A, *p < 0.01) accompanied by a clinical score (Additional file 1, Figure S1A). Body mass did not change between treatment and vehicle group (Additional file 1, Figure S1B). Early model optimization data was collected but is not shown here for clarity. Serum cytokine levels were inconsistent and in many cases undetectable as expected (data not shown). However, using this extraction method both early (Figure 2B, IL-1β) and late (Figure 2C, IL-12) cytokines could be detected. Cytokine IL-1β, one of the signature cytokines of RA and a target of a commonly used RA therapeutic [19,20], is found shortly after arthritis induction and can be reduced upon dexamethasone treatment (Figure 2B, *p < 0.05). Similar findings were found for IL-6 and IL-1β using conventional RT-PCR methods (data not shown). The source for IL-1β are usually activated macrophage, dendritic cells and NK cells [21]. The source for IL-12 are usually tissue activated macrophage (F4/80^+^) and dendritic cells but can also be made by B-cells [22,23]. IL-12 is usually observed following early innate cytokines including TNFα and IL-6 [24,25]. Cytokines TNFα and IL-2 were also reduced upon dexamethasone treatment (Additional file 1, Figure S1C-D). This reduction in inflammatory cytokines corresponded with reduced inflammation and cartilage degradation as demonstrated in paw histological H&E and Safranin-O sections (Figure 2D).

**Figure 3:**
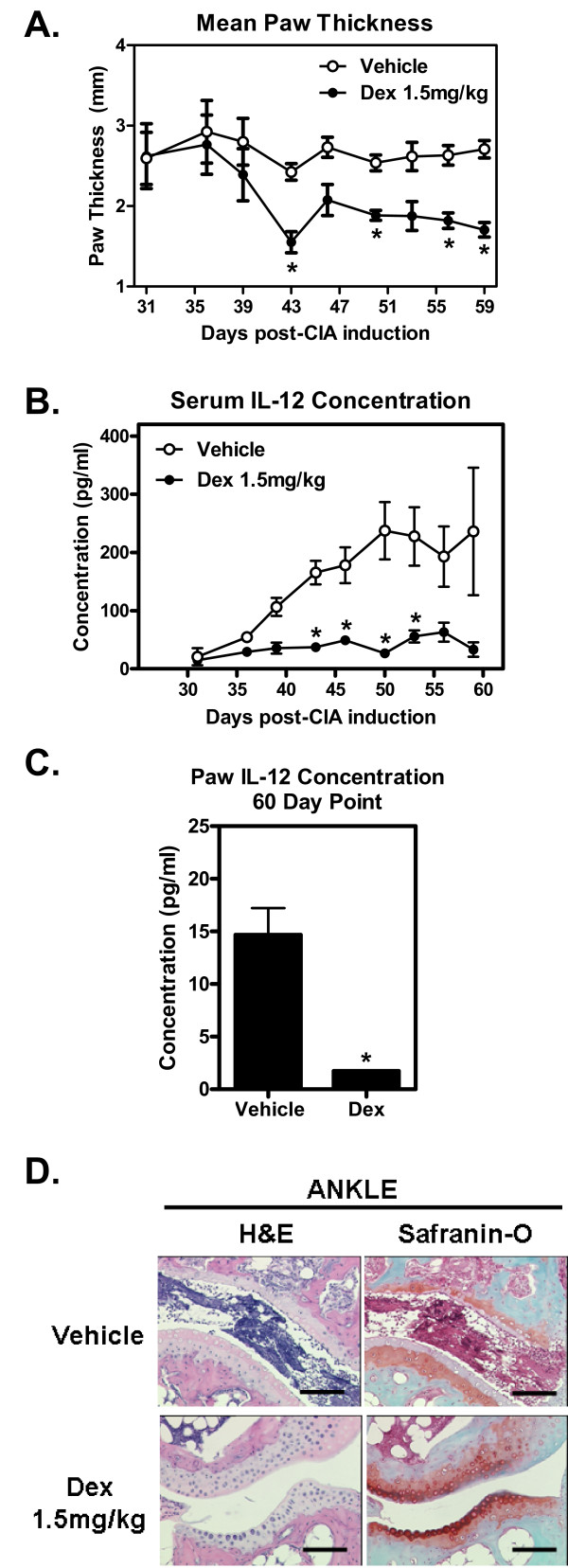
**Paw Cytokines Follow Disease Progression in CIA Mouse Model**. Female DBA/1 mice were injected i.d. at the base of the tail with collagen II plus CFA, followed by a booster s.c. on day 21 with IFA followed by a 28 day LPS priming, full onset of arthritis was between 30-35 days. **(A) **Disease is established and mice enter the study when specific criteria are met (see materials and methods). Dexamethasone treatment at 1.5 mg/kg given i.p. three times a week significantly reduced paw thickness (average from independently measured mice) compared to vehicle control mice, *p < 0.05, N = 10 mice per group. **(B) **Serum IL-12 levels were significantly reduced upon dexamethasone treatment as compared to vehicle control group, *p < 0.05. **(C) **Paw IL-12 levels were assessed by our Luminex^® ^extraction protocol. IL-12 levels measured at the final time point were significantly less in the dexamethasone treated mice as expected from reduced serum IL-12 levels, *p < 0.05. **(D) **Histopathology H&E and Safranin-O representative slides from five mice (tarsus shown) at 4x magnification show that dexamethasone treated mice have reduced inflammation, matrix degeneration and joint disfiguration compared to the control vehicle mouse. All graphs show Mean ± SEM.

This extraction method also works for the more conventional CIA model where chronic arthritis provides an array of cytokines that can be monitored both in the peripheral circulation but also locally in the paw. Mice immunized against collagen type II induce an inflammatory cascade that is triggered and propagated by the typical Th1/Th17-cellular response and involves cytokines IL-6, IL-1β, TNFα, GM-CSF and IL-12 [26]. Disease can be treated with the general standard-of-care, dexamethasone, providing reduced paw swelling (Figure 3A), and clinical score (Additional file 2, Figure S2A) without overt toxicity to the animal as shown by stable body mass (Additional file 2, Figure S2B). Reduction of circulating serum anti-collagen type II antibodies was not observed nor correlated with disease treatment suggesting that dexamethasone only treats the symptoms and not the actual propagator of this disease (Additional file 3, Figure S3). A significant drop in circulating serum IL-12 using dexamethasone (Figure 3B) corresponds with a reduced 60 day IL-12 level in the paws of affected animals (Figure 3C). Similar results were observed for spleen (data not shown). In addition, consistent drop in IFNγ and IL-6 upon dexamethasone treatment (data not shown) was also observed. The CIA model recapitulates many of the common cytokine cascades that support disease augmentation and using our method we were able to monitor cytokine levels increasing and decreasing overtime in the paw in a much more dynamic manner than observed in the serum (Additional file 4, Figure S4). As expected IL-1β starts low at the beginning of disease and as disease progresses IL-1β levels surge only to drop later as the disease begins to resolve (Additional file 4, Figure S4). A spike of IL-10 is observed early in disease possibly due to latent activated macrophage [27] followed by a consistent elevated level of IL-6 cytokine as expected [28]. An unlikely result is the low expression of GM-CSF and TNFα in the paw of CIA mice (Additional file 4, Figure S4). Histology results mirror expected responses as indicated by the high levels of cytokines found in the paw and serum further validating our paw cytokine extraction method (Figure 3D).

### Sensitive Detection of Phospho-STAT3 Activation in Paws of Arthritic Mice

What is better than the detection of extracellular molecules involved in driving the deleterious immune response liable for observed tissue damage is actually monitoring and measuring the levels of signaling molecules and transcription factors involved in generating or supporting these cytokine cascades. Measuring STAT proteins necessary to transduce signals from several cytokine receptors such as IFNγR (e.g. STAT1), IL6R (e.g. STAT3) and IL-2Rβ/γ_c _(e.g. STAT5a/b) provides the investigator with a way of monitoring developing disease activity and can better support drug discovery efforts by homing in on key cascades. However, it is important to note that several different cytokine receptors may converge on a single STAT transcription factor pathway and are not limited to these particular STATs and the converse is true that a single cytokine receptor may activate more than one STAT [29].

**Figure 4:**
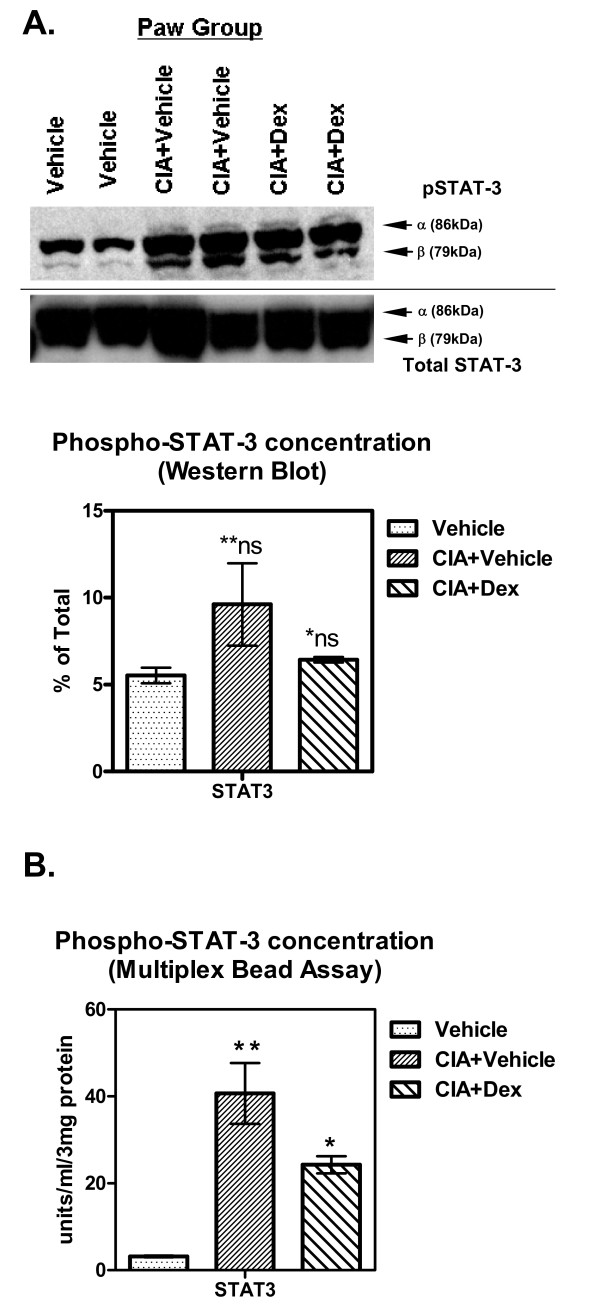
**Using Luminex^® ^Multiplex Bead Arrays to Measure Intracellular PD Markers is More Sensitive than Conventional Western Blot Analysis**. Using the 60 day CIA mouse paws we extracted the paw supernatants as described in the materials and methods and tested for phospho-STAT3 using either conventional western blot analysis with densitometry or multiplex bead analysis. **(A) **Extracts from two different mice per group were run on a SDS-PAGE gel and transferred onto a nitrocellulose membrane and exposed to film for the final image. Western blot was probed with anti-pSTAT3 α (79 kDa) and β (86 kDa) subunits, stripped then re-probed for total STAT3, α (76 kDα) and β (86 kDa) subunits as shown. Below graph shows densitometry analysis from above blot **ns p = 0.2037 versus vehicle, *ns p = 0.1226 versus CIA + vehicle group, N = 3 per group shown. **(B) **Luminex^® ^phospho-STAT kit analyzed paw extractions from CIA mice treated with vehicle alone or dexamethasone. Samples were those same as used for the W.blot above, *p = 0.034 compared CIA + vehicle group, **p = 0.017 compared to vehicle alone. For both graphs, statistical test used for p-values: two-tailed paired Student's t-test, N = 3-4 mice per group shown. All graphs show Mean ± SEM, ns = not significant.

To compare our multiplex bead method to the conventional western blot analysis we ran inflamed paw extracts from CAIA mice on a western blot side-by-side with the multiplex bead assay measuring phosphorylated STAT1 (pSTAT1), STAT3 (pSTAT3) and STAT5a/b (pSTAT5a/b). Both pSTAT1 and pSTAT5a/b were similar to the vehicle only group (data not shown), however, pSTAT3 showed a dramatic change from vehicle control group and this was similar with results seen for the western blot (Figure 4A-B; only pSTAT3 data shown). Lanes represent two independent samples from each group, densitometry analysis results are shown below the blot. Comparing these results (Figure 4A) to the multiplex bead results (Figure 4B) the bead array provides better data resolution and a lower and more reproducible background. Although, results are comparable between the two methods, the Luminex^® ^data provides greater statistical significance due to the larger signal to noise ratio. However, the bead array provides the ability to simultaneously measure several different STAT molecules in a single high-throughput manner. Similar results were observed for the spleen tissue but were not as robust as the paw where most of the disease activity is confined (data not shown).

**Figure 5:**
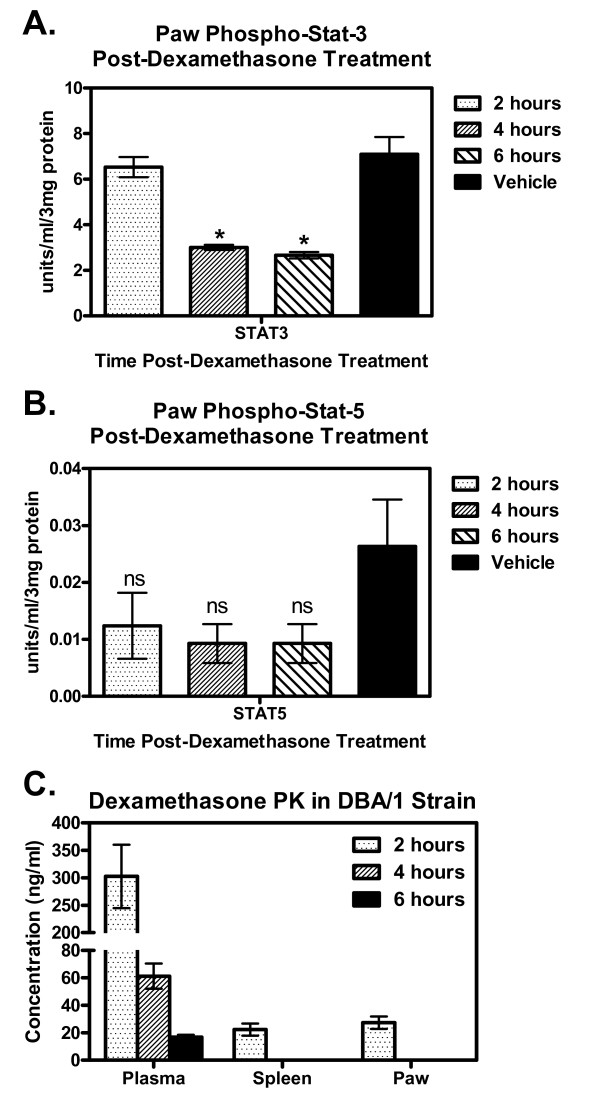
**Specific Reduction of pSTAT3, but not pSTAT5 in Dexamethasone Treated CAIA Mice Overtime Corresponds with Local Dexamethasone Pharmacokinetics**. Using our CIA mouse model of RA we treated 35 day mice with either vehicle alone or dexamethasone and looked overtime at the loss of pSTAT3 and pSTAT5 in the paws using our extraction method. **(A) **Paws from treated mice were processed and analyzed using Luminex^® ^technology, rapid decrease in pSTAT3 observed within 4 hours post dexamethasone treatment and remains decreased as shown for the 6 hour time point compared to vehicle alone, *p < 0.05. **(B) **No significant change observed for pSTAT5, trend decrease in overall pSTAT5 levels, ns = not significant as compared to vehicle alone. **(C) **PK of dexamethasone in the plasma, spleen and paw extracts from treated mice, see materials and methods for complete methods for PK analysis. All graphs show Mean ± SEM, N = 4 mice per group.

Using the CAIA model significant decrease in the level of pSTAT3 as early as 4 hours after dexamethasone treatment (Figure 5A) could be observed, however no significant change in pSTAT5a/b levels were ever observed, albeit a trend decrease does support the case of dexamethasone acting as a general immunosuppressant (Figure 5B). As shown by pharmacokinetic analysis, dexamethasone can be detected in the paw as early as 2 hours post-injection and can affect downstream effector cascades (Figure 5C). This suggests that similar therapies can be used with this reference standard of care (SoC) agent to monitor cytokine pathway modulation in conjunction with disease as exemplified in Figures 2.0 and 3.0. Similar pSTAT results were also obtained for the CIA model (Additional file 5, Figure S5) for both pSTAT3 and pSTAT5, no change in pSTAT1 was observed in any model tested (data not shown).

## Discussion

Here we discuss a novel, yet simple, method to extract tissue cytokines and transcription factors from the paws of mice with rheumatoid arthritis. Cytokines play a major role in the development of arthritis, especially at the site of initial insult, the synovial joint. *Ex vivo *end analyses of sectioned joints only give a snap shot of active cytokine and signaling pathways and many antigens are destroyed in the processing of these tissues. Our extraction method provides the investigator with a means to measure native proteins in their active confirmation without the addition of exogenous treatments. In addition, using various phosphatase and protease inhibitors we are able to detect endogenously activated phosphorylated STATs that correlate well with disease activity and treatment. This method will be useful for following treatment with a small molecule inhibitor or biologic modulating cytokine pathways.

Western blots are extremely useful for the detection of trace proteins; however, one of the major limitations is time and the ability to multiplex. With the advent of fluorescent western blots multiple proteins can be observed on a single membrane using different fluorochromes and excitation channels. Quantitative RT-PCR is also an option; however, for the average drug discovery scientist running hundreds of *ex vivo *samples becomes tedious and time consuming. Multiplex bead array technology has advanced to provide the researcher with a multitude of options. In addition, to detecting phospho-STAT proteins we have recently expanded to include the detection of signaling molecules such as Akt and protein kinase C (PKC), also molecules involved in the immuno-proteasome pathway. This means that it is possible to use this extraction method coupled with multiplex bead arrays for different disease models. One example is to use this method for studying lupus arthritis in the MRL/lpr mouse model for rapidly developing lymphoproliferative disease [30]. Several phenotypes of human systemic lupus erythematosus (SLE) are reproduced in the commonly used New Zealand Merged (NZM) and MRL/lpr (Fas^lpr^) models [31,32]. Dermatitis, lupus arthritis and lupus nephritis are some of the most common lupus-related symptoms observed in these systems [33]. Thus a possible alternative use for this extraction method is the ability to measure various cytokines and signaling molecules within the same sample from all three affected regions of the lupus-prone animal - joints (lupus arthritis), skin (dermatitis) and kidneys (lupus nephritis).

Paw swelling is slowly reduced by dexamethasone in the CAIA model as expected, however, paw IL-1β and IL-12 does not significantly decrease below vehicle until days 9 and 10 respectively (Figure 2). Since paw swelling is significantly reduced below that of vehicle by day 9 it is expected that this reduction is in due part to these reduced cytokine levels. The molecular signaling events for this change can be observed using both Western blot and Luminex^® ^cytokine beads, however, due to the lowered sensitivity of Western blots a significant result using the multiplex bead method is lost, using Western blots, due to signal strength heterogeneity (Figure 4).

Many of the expected cytokines are raised during arthritis in this model, such as IL-1β, IL-10 and IL-6, however, unexpectedly both GM-CSF and TNFα remain low (Additional file 4, Figure S4). GM-CSF being a survival and growth factor for several haematopoietic cells especially for granulocytes and monocytes, we would expect an increase in paw expression overtime. Exogenous GM-CSF and TNFα have both been shown to induce arthritis in mice [34,35]. This particular study provides a limited view and is intended to give the reader an idea of how paw cytokines could be followed overtime using this multiplex technology and is not intended to be a guide. Upon multiple repeats we would expect some heterogeneity but that both TNFα and GM-CSF should remain elevated in the swollen paw.

Another unexpected result was the failure of dexamethasone treatment to reduce the levels of circulating anti-collagen type II (CII) antibodies below that of vehicle (Additional file 3, Figure S3). High dose dexamethasone can induce lymphocyte apoptosis, hence the use of high dose dexamethasone for the treatment of multiple myeloma [36]. This failure to reduce anti-CII antibodies can be explained by the fact that mouse IgG has a very long plasma half-life (t_1/2_~28 days). Dexamethasone treatment of paw swelling may also reduce the levels of circulating antibody secreting cell types but impact on circulating anti-CII IgG may not be observed until several months post-treatment.

One consistent observation that was noticed between the models was the general lack of pSTAT5a/b or pSTAT1 activation over vehicle alone (data not shown). All three STAT molecules were measured including STAT5a/b, STAT1 and STAT3. STAT molecules are becoming more popular with their essential role in autoimmunity, cancer and even central nervous system (CNS) disorders [6,37-39]. STAT molecules are unique in that they can act as signaling molecules, transcription factors, have been shown to interact with NFκB and stabilize performance and now there is accumulating evidence that there are mitochondrial STAT molecules responsible for interacting with the electron-transport system and adenosine triphosphate (ATP) synthesis via interaction with Ras [38,40,41]. As for our observations, in these models, we would have expected to see STAT3, STAT-4, STAT5a/b and STAT1 activation as these molecules are downstream of the IL-6 (IL-6α/GP130), IL-12 (IL-12Rβ), GM-CSF (GM-CSFRa/b), and IFNγ (IFNγR1/2) receptors respectively. The presence of IL-12 in the paws was not surprisingly unusual as the CIA model is mainly driven by typical Th1 responses and IL-12 helps support the signal for T-bet expression and Th1 cell differentiation [42-44]. Cytokines IFNγ and IL-6 also increased with disease and could be reduced upon dexamethasone treatment (data not shown). It is quite possible that the source of IFNγ are Th1 cells or NK cells while IL-6 is most likely provided by activated macrophage. It is this IL-6 autocrine cycle that is most probably responsible for the high levels of pSTAT3 observed in the paws of affected animals. The ability of dexamethasone to decrease intrinsic pSTAT3 levels and several cytokines is most likely due to simply less inflammation as observed by reduced mean paw size and clinical score. Dexamethasone, member of the glucocorticoid steroid family, is known to work via several mechanisms including the rapid upregulation of new IκBα preventing NFκB activation and nuclear translocation [45], eventually leading to immunosuppression. Dexamethasone is commonly used to treat various autoimmune indications including rheumatoid arthritis (RA) and SLE; in addition dexamethasone may also be used to treat various hematological malignancies [46-48].

As expected, the two models produced similar but not completely overlapping cytokine cascades in the affected paws. The CAIA model is more dependent on the actions of tissue antibody-dependent cell cytotoxicity (ADCC) and that of macrophage and neutrophils; early innate cytokines dominate such as IL-1β and TNFα (figure 2 and data not shown). Cytokine IL-12 levels slowly increase as macrophage and dendritic cells (DCs) mature with disease progression (Figure 2C). As for the CIA model which is more dependent on typical Th1/Th17 responses further activating synovial macrophage, high levels of paw IL-12 (Figure 3C) along with the classical early innate cytokines such as IL-1β (Additional file 5, Figure S5A) and IL-6 (Additional file 5, Figure S5A and B) are also observed.

## Conclusions

In summary, we have developed a novel method of extracting and protecting cytokines and related signaling molecules from degradation and coupled it to multiplex bead arrays where by multiple potential biomarkers can be monitored simultaneously from the same sample directly *ex vivo*. This method is more streamlined and time-efficient than several conventional methods such as western blot analysis or RT-PCR, measures molecules in their native, non-reduced form, and can be coupled to *ex vivo *pharmacodynamics (PD) and pharmacokinetic (PK) analyses. This method may be applied to lupus models, various different organ types, such as the kidneys, and even tumor xenograft models. This method will help contribute to the understanding of how dynamic cytokine cascades at the site of disease in arthritis may aid in the discovery of new compounds or biologics with the potential to modulate local deleterious immune responses.

## Abbreviations

(CIA): Collagen-induced arthritis; (CAIA): Collagen-antibody induced arthritis; (RA): Rheumatoid Arthritis; (CII): Type II Collagen

## Authors' contributions

LL performed all *ex vivo *assays and participated in the production of this manuscript, KS performed most of the *in vivo *work and developed both the CIA and CAIA models, MS was the senior scientist on this project and lead the design, optimization and validation of both arthritis models and was the primary author for this manuscript. All authors approved the final manuscript.

## Supplementary Material

Additional file 1 **Clinical Score and Body Mass of CAIA Mice Treated with Dexamethasone**. Female DBA/1 mice injected with arthritogenic antibodies and LPS develop RA and begin treatment by day 6. **(A) **Total clinical score of mice treated with vehicle or dexamethasone, three times a week. Clinical score definitions can be found in the materials and methods. N = 10 per group, *p < 0.001, Mann-Whitney, two-tailed test. **(B) **Mean body mass of all mice treated, no significant difference observed between any group, N = 10 mice per group. All graphs show Mean ± SEM. **(C) **Paw IL-2 and **(D) **TNFα concentration sampled in 3 mg of total protein prepared as described in the materials and methods *p < 0.05. Click here for file

Additional file 2 **Clinical Score and Body Mass of CIA Mice Treated with Dexamethasone**. Female DBA/1 mice were induced to develop CII-dependent arthritis using purified bovine CII with CFA and boosted with CII in IFA, then later primed with LPS to provide an acute local response in the joints of affected mice by day 30 after initial immunization. **(A) **Total clinical score shown, dexamethasone treatment was provided three times a week at 1.5 mg/kg, *p < 0.01, Mann-Whitney, two-tailed test, N = 10 mice per group. **(B) **Mean body mass of all mice treated, no significant difference observed between any group, N = 10 mice per group. All graphs show Mean ± SEM. Click here for file

Additional file 3 **Serum Levels of Anti-Collagen type II Autoantibodies in CIA Model**. Serum IgG anti-collagen type II autoantibodies were measured by ELISA as described in the materials and methods. No significant change was observed at any time point, N = 4 mice shown, graph shows Mean ± SEM. Click here for file

Additional file 4 **Paw Cytokine Levels in the CIA Model during Disease Development**. Female DBA/1 mice were induced to develop CII-dependent arthritis using purified bovine CII with CFA and boosted with CII in IFA, then later primed with LPS to provide an acute local response in the joints of affected mice by day 30 after initial immunization. Age matched female DBA/1 mice were either not induced (0 wk time point) or induced to develop CIA by week 4. Paws from each week were removed from CIA mice and cytokines were measured using our extraction method coupled with Luminex^® ^to generate the graph shown. Click here for file

Additional file 5 **Specific Reduction of pSTAT3 and not pSTAT5 in Dexamethasone Treated CIA Mice**. Sixty day inflammed CIA mouse paws were processed and analyzed using Luminex^® ^kits for the presence of activated pSTAT3 and pSTAT5. **(A) **Reduction in paw levels of pSTAT3 observed after three rounds of Dexamethasone treatment, 1.5 mg/kg, *p < 0.05. **(B) **Lack of pSTAT5 reduction observed in dexamethasone treated mice similar to what is observed in the CIA model. All graphs show Mean ± SEM, N = 3-4 per group. Click here for file
