# Supplementary figures and images for: Novel Method of Monitoring Trace Cytokines and Activated STAT Molecules in the Paws of Arthritic Mice using Multiplex Bead Technology
Source: BMC Immunol. 2010 Nov 12;11:55. doi: 10.1186/1471-2172-11-55 (PMC2992046; doi:10.1186/1471-2172-11-55)

**A.**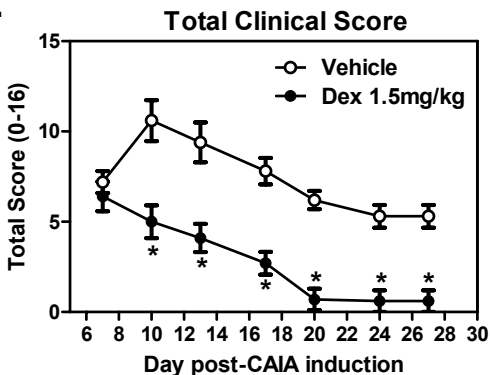**B.**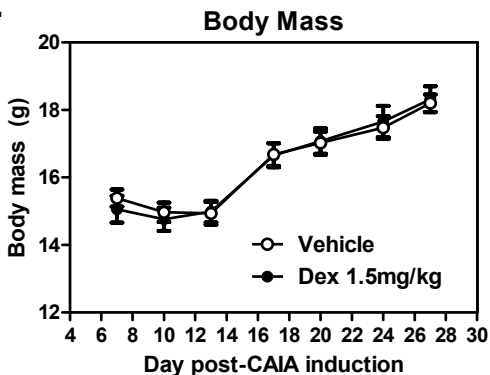**C.**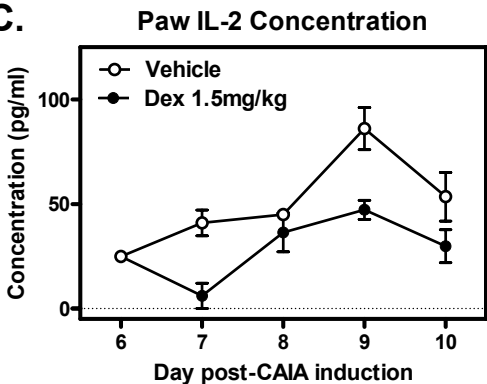**D.**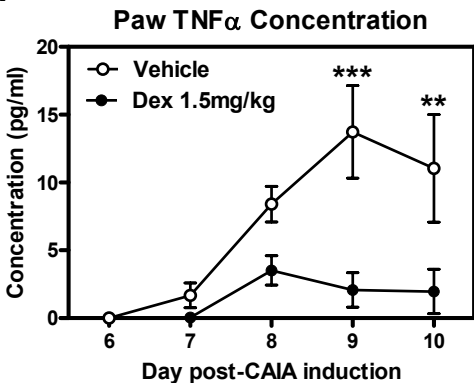

Supplement: Additional file 1 — Clinical Score and Body Mass of CAIA Mice Treated with Dexamethasone. Female DBA/1 mice injected with arthritogenic antibodies and LPS develop RA and begin treatment by day 6. (A) Total clinical score of mice treated with vehicle or dexamethasone, three times a week. Clinical score definitions can be found in the materials and methods. N = 10 per group, *p < 0.001, Mann-Whitney, two-tailed test. (B) Mean body mass of all mice treated, no significant difference observed between any group, N = 10 mice per group. All graphs show Mean ± SEM. (C) Paw IL-2 and (D) TNFα concentration sampled in 3 mg of total protein prepared as described in the materials and methods *p < 0.05. [file 1471-2172-11-55-S1.PDF]

**A.**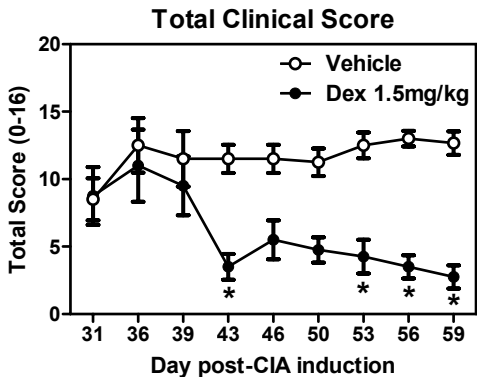**B.**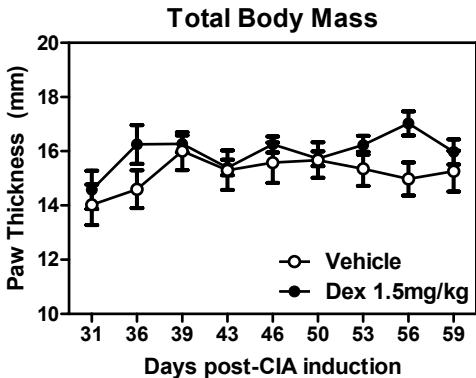

Supplement: Additional file 2 — Clinical Score and Body Mass of CIA Mice Treated with Dexamethasone. Female DBA/1 mice were induced to develop CII-dependent arthritis using purified bovine CII with CFA and boosted with CII in IFA, then later primed with LPS to provide an acute local response in the joints of affected mice by day 30 after initial immunization. (A) Total clinical score shown, dexamethasone treatment was provided three times a week at 1.5 mg/kg, *p < 0.01, Mann-Whitney, two-tailed test, N = 10 mice per group. (B) Mean body mass of all mice treated, no significant difference observed between any group, N = 10 mice per group. All graphs show Mean ± SEM. [file 1471-2172-11-55-S2.PDF]

# Serum Anti-Collagen II Antibody

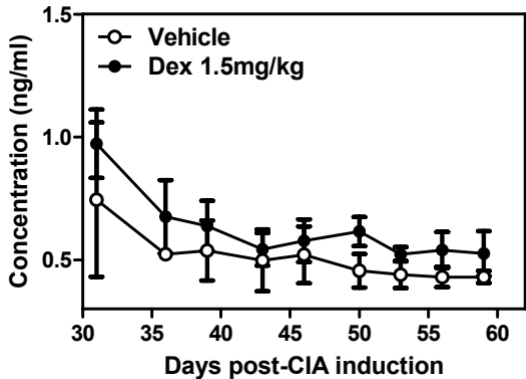

Supplement: Additional file 3 — Serum Levels of Anti-Collagen type II Autoantibodies in CIA Model. Serum IgG anti-collagen type II autoantibodies were measured by ELISA as described in the materials and methods. No significant change was observed at any time point, N = 4 mice shown, graph shows Mean ± SEM. [file 1471-2172-11-55-S3.PDF]

# Paw Cytokine Concentration

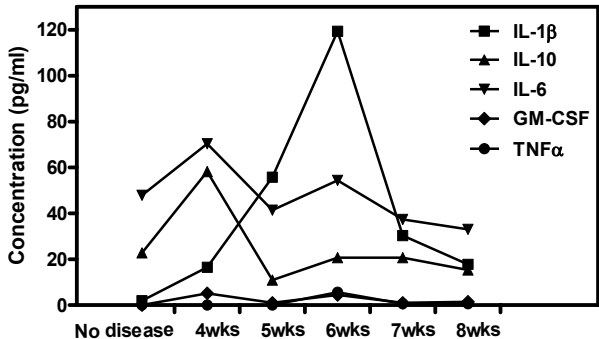

Supplement: Additional file 4 — Paw Cytokine Levels in the CIA Model during Disease Development. Female DBA/1 mice were induced to develop CII-dependent arthritis using purified bovine CII with CFA and boosted with CII in IFA, then later primed with LPS to provide an acute local response in the joints of affected mice by day 30 after initial immunization. Age matched female DBA/1 mice were either not induced (0 wk time point) or induced to develop CIA by week 4. Paws from each week were removed from CIA mice and cytokines were measured using our extraction method coupled with Luminex® to generate the graph shown. [file 1471-2172-11-55-S4.PDF]
