# Supplementary material for: Novel Method of Monitoring Trace Cytokines and Activated STAT Molecules in the Paws of Arthritic Mice using Multiplex Bead Technology
Source: BMC Immunol. 2010 Nov 12;11:55. doi: 10.1186/1471-2172-11-55 (PMC2992046; doi:10.1186/1471-2172-11-55)
Supplement: Additional file 5 — Specific Reduction of pSTAT3 and not pSTAT5 in Dexamethasone Treated CIA Mice. Sixty day inflammed CIA mouse paws were processed and analyzed using Luminex® kits for the presence of activated pSTAT3 and pSTAT5. (A) Reduction in paw levels of pSTAT3 observed after three rounds of Dexamethasone treatment, 1.5 mg/kg, *p < 0.05. (B) Lack of pSTAT5 reduction observed in dexamethasone treated mice similar to what is observed in the CIA model. All graphs show Mean ± SEM, N = 3-4 per group. [file 1471-2172-11-55-S5.PDF]

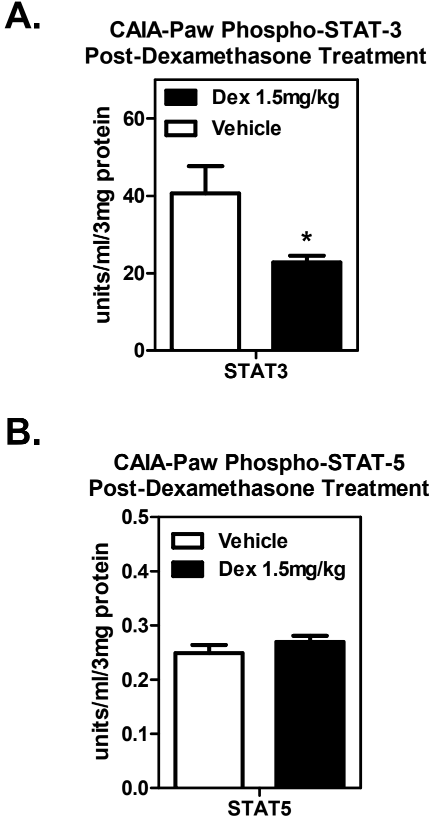
A.
CAIA-Paw Phospho-STAT-3
Post-Dexamethasone Treatment
Dex 1.5mg/kg
Vehicle
units/ml/3mg protein
60
40
20
0
*
STAT3
B.
CAIA-Paw Phospho-STAT-5
Post-Dexamethasone Treatment
Vehicle
Dex 1.5mg/kg
units/ml/3mg protein
0.5
0.4
0.3
0.2
0.1
0.0
STAT5
